# Comprehensive transcriptomic and proteomic characterization of human mesenchymal stem cells reveals source specific cellular markers

**DOI:** 10.1038/srep21507

**Published:** 2016-02-09

**Authors:** Anja M. Billing, Hisham Ben Hamidane, Shaima S. Dib, Richard J. Cotton, Aditya M. Bhagwat, Pankaj Kumar, Shahina Hayat, Noha A. Yousri, Neha Goswami, Karsten Suhre, Arash Rafii, Johannes Graumann

**Affiliations:** 1 Research Division, Weill Cornell Medicine-Qatar, Doha, State of Qatar

## Abstract

Mesenchymal stem cells (MSC) are multipotent cells with great potential in therapy, reflected by more than 500 MSC-based clinical trials registered with the NIH. MSC are derived from multiple tissues but require invasive harvesting and imply donor-to-donor variability. Embryonic stem cell-derived MSC (ESC-MSC) may provide an alternative, but how similar they are to *ex vivo* MSC is unknown. Here we performed an in depth characterization of human ESC-MSC, comparing them to human bone marrow-derived MSC (BM-MSC) as well as human embryonic stem cells (hESC) by transcriptomics (RNA-seq) and quantitative proteomics (nanoLC-MS/MS using SILAC). Data integration highlighted and validated a central role of vesicle-mediated transport and exosomes in MSC biology and also demonstrated, through enrichment analysis, their versatility and broad application potential. Particular emphasis was placed on comparing profiles between ESC-MSC and BM-MSC and assessing their equivalency. Data presented here shows that differences between ESC-MSC and BM-MSC are similar in magnitude to those reported for MSC of different origin and the former may thus represent an alternative source for therapeutic applications. Finally, we report an unprecedented coverage of MSC CD markers, as well as membrane associated proteins which may benefit immunofluorescence-based applications and contribute to a refined molecular description of MSC.

Mesenchymal stem cells (MSC) are self-renewing multipotent cells which hold great potential in regenerative medicine and tissue engineering. Their versatility, homing potential to injured tissue, and the limited risk of tumorigenesis^1^ render them an interesting tool in cell-based personalized therapy. Leveraging differentiation ability, strong immunomodulatory capacity and paracrine support, they have been used in the experimental treatment of a plethora of diseases, evident in the more than 500 active MSC-based clinical trials worldwide currently registered with the NIH. Studies have shown that treatment with MSC is beneficial to patients with Graft versus Host Disease^2^,^3^, autoimmune diseases^4^,^5^,^6^, cardiovascular^7^ and liver disease^8^. In MSC-based therapies, the actual engraftment of MSC is considered to be very low and not persistent^9^,^10^. Tissue regeneration is initiated and supported by MSC at the site of tissue damage mainly by immunomodulation, through both cell-free (secretion) or cell-based mechanisms. Importantly, allogenic MSC do not induce a strong host response due to their low expression of MHC class II molecules and lack of co-stimulatory receptors, rendering them transplantable with comparative ease^11^.

MSC were first isolated from bone-marrow^12^ but actually reside perivascular in a wide range of organs and tissues such as adipose tissue, skin, muscle, tendon, lungs, dental pulp, umbilical cord, placenta, with each population displaying individual differentiation potential and phenotype^13^,^14^,^15^. In general, MSC can be differentiated *in vitro* into cells of the mesodermal lineage such as osteocytes, adipocytes and chondrocytes^16^,^17^. A smaller number of studies have also shown differentiation into myocytes, cardiomyocytes and even transdifferentiation into cells of the endoderm or ectoderm^17^,^18^. Adult tissue derived MSCs can be expanded *in vitro* but lose proliferation, differentiation and immunomodulation potential gradually over time^18^,^19^. Recently described embryonic stem cell-derived MSC (ESC-MSC), however, are highly proliferative and an interesting source for clinical use. ESC-MSC carry typical MSC markers and have been shown to have similar *in vitro* and *in vivo* capabilities as BM-MSC, including homing, immunomodulation, regeneration and differentiation^20^,^21^,^22^,^23^,^24^,^25^,^26^ . One *in vivo* study even showed the superiority of ESC-MSC in comparison to BM-MSC in the treatment of an experimental autoimmune encephalomyelitis (EAE) model of Multiple Sclerosis^27^.

To date, no surface markers have been exclusively associated with MSC. Based on a proposal by the International Society for Cell Therapy, cells may be classified as MSC if they adhere to plastic, carry a minimal subset of characteristic surface markers (CD73, CD90, CD105), are negative for hematopoietic lineage markers (CD14, CD19, CD45) and present the potential to differentiate into bone, fat and cartilage^28^.

For the development of targeted MSC-based therapy, an unambiguous definition of MSC is highly desirable and comprehensive molecular characterizations, as delivered by “Omics” methodologies, may be one approach on the path towards that goal^29^.

Given their donor-derived nature and limited expansion capacity in cell culture, the production of adult MSC at the scale required for efficient therapeutic use is challenging. MSC derived from embryonic stem cells (ESC-derived MSC; ESC-MSC), in comparison, are an alternative source with additional potential benefits with respect to biosafety^30^. A future goal may be the use of induced pluripotent stem cells to produce personalized MSC at large scale prior to treatment.

Establishing molecular equivalency (or the lack thereof) between differently sourced MSC is a central question for future clinical use of ESC-MSC. We present a comprehensive transcriptomic and proteomic characterization of ESC-MSC and their comparison to bone marrow-derived MSC as well as to embryonic stem cells (ESC). The ESC-MSC used were derived through a protocol potentially adaptable to clinical use, which enriches a uniform MSC population from spontaneously differentiated ESC^31^. The rationale for selecting this method was based on its simplicity, robustness and the adaptability to clinical applications. To our knowledge, this is the first large-scale proteomic characterization of ESC-MSC. Two global profiling techniques were combined for a comprehensive analysis: RNA deep sequencing as well as quantitative high resolution nano-LC-MS/MS based on stable isotope labeling with amino acid in cell culture (SILAC)^32^. This extensive comparison delivers unprecedented coverage of cluster of differentiation (CD) markers, which may contribute to a more stringent definition of MSC as a cell type.

## Methods

### Cell culture and SILAC labeling of embryonic stem cells (ESC) and mesenchymal stem cells (ESC-MSC, BM-MSC)

ESC-derived MSC were generated according to a published protocol^31^. Briefly, ESC seeded in 6-well plates were cultured under feeder-free condition on growth factor reduced Matrigel (BD Invitrogen) with daily changes of mTeSR1 medium (Stem Cell Technologies) supplemented with 1% penicillin/streptomycin. Three days after passage with accutase, medium was changed to DMEM/low glucose supplemented with 10% FBS, GlutaMax and 1% penicillin/streptomycin. Cells were left undisturbed for 6 days with one medium change after 3 days. Differentiating ESC were passaged 1:3 using trypsin and seeded on plastic. After another 6 days of culture with one medium change after three days, cells were passaged again with trypsin and seeded on plastic. From this passage onwards, cells are fully differentiated into ESC-MSC. ESC-derived MSC have been phenotyped and show classic MSC behavior. They are plastic-adherent, show trilineage differentiation and express MSC-typical surface markers (CD73, CD90, CD105)^31^. ESC-MSC used for transcriptomic and proteomic profiling were generated in three independent experiments. Bone marrow-derived mesenchymal stem cells (Lonza, StemCell Technologies) were purchased with their mesenchymal characteristics verified. Details of the BM-MSC donors: 40y/m (StemCell, MSC-001F, lot#BM2893), 39y/m (Lonza, PT2505, lot#1F3422), 27y/m (Lonza, PT2505, lot#318006), 20y/m (Lonza, PT2505, lot#8F3520). Since the origin ESC line is male, only male BM-MSC donors were selected. For proteomic and transcriptomic profiling, BM-MSC of three (40y, 39y, 27y) and four different donors (40y, 39y, 27y, 20y) were included, respectively. Representative pictures for ESC, ESC-MSC and BM-MSC are shown (Figure S1A). All cells were cultured at 37 °C in an atmosphere supplemented to 5% CO_2_.

Permission to use the human embryonic stem cell line (hESC) ES04 (WiCell institute) was obtained from the Cornell/Rockefeller/Sloan Kettering tri-institutional ESC research oversight committee. Funding was secured from nonfederal, US-external funding sources. ESCs were maintained under feeder-free condition on growth factor reduced Matrigel (BD Invitrogen) in mTeSR1 medium (Stem cell technologies) supplemented with 1% (v/v) penicillin/streptomycin. Medium was changed daily. ESC-MSC and BM-MSC were maintained in DMEM low glucose supplemented with 10% FBS, GlutaMax and 1% penicillin/streptomycin. ESC-MSC were sub-cultured every 3–4 days, BM-MSC every 7 days. Cells were harvested at 80% confluence at the following passages: ESC at passages 89–91, BM-MSC at passages 5–6 and ESC-MSC at passages 7–9.

SILAC labeling was performed as described^33^. Arginine/lysine-free medium (custom made-mTeSR1 for ESC, DMEM low glucose with dialyzed FBS for MSC) was supplemented either with L-lysine – U-^13^C_6_^15^N_2_ (Lys8) and L-arginine – U-^13^C_6_^15^N_4_ (Arg10) for “heavy” labeling, or with L-lysine – U-^13^C_4_^15^N_0_ (Lys4) and L-arginine – U-^13^C_6_^15^N_0_ (Arg6) for “medium” labeling, or with standard amino acids for “light” labeling at a concentration matching the original medium. Labeled amino acids were purchased from Cambridge Isotope Laboratories. Cells were labeled for >5 doublings and incorporation was verified prior to sample mixing. Labeled protein samples were combined as shown in Figure S4A.

### Immunoblotting

Proteins (5 μg) were separated by SDS-PAGE (Bis-Tris NuPAGE gradient gels 4–12%, Lifescience technologies) and transferred onto PVDF membranes (HybondLF, GE Healthcare). The membranes were blocked in TBS/0.05% Tween20 containing 3% BSA and were incubated overnight with primary antibodies (Cell Signaling: αLSD1 #2139, αMEK1/2 #4694, αhistone H2A.Z #2718) followed by horse radish peroxidase conjugated secondary antibodies (SantaCruz Technologies: α-rabbit #sc-2317, α-mouse #sc-2314, Promega: α-mouse #W4028). Membranes were incubated with the ECL™ Prime Western Blotting Detection Reagent (GE Healthcare) and chemiluminescence was detected with a CCD camera (SnapGene v.7.04).

### Next generation RNA sequencing

Total RNA was extracted with TRIZOL and further cleaned on RNAeasy columns (Qiagen). RNA quality was evaluated by the Agilent Bioanalyzer 2100 system. Only preparations with RIN (RNA integrity number) values above 8 were considered.

Following RNA isolation, 100 ng of total RNA was converted to cDNA using the Ovation RNA-seq System V2 (Nugen Technologies, San Carlos, CA). 2 μg of the amplified cDNA was sheared to 150–200 bp size distribution by Adaptive Focused Acoustics using a Covaris E220 instrument (Covaris, Woburn, MA) under the following conditions: 50 μl total volume, 20% duty cycle, 175 intensity, 200 cycles per burst, 165 sec in frequency sweeping mode. The remainder of the library preparation followed manufacturer’s protocol as described in Encore Rapid IL Multiplex System (Nugen Technologies). Briefly, the sheared cDNA was end-repaired to generate blunt ends then ligated to Illumina compatible adaptors with indexing tags, followed by 1× AMPure XP beads purification. The final NGS libraries were quantified using Agilent Bioanalyzer DNA Chip 1000 then pooled; 11 libraries per pool. Paired-end 100 bp deep sequencing was carried out on HiSeq 2500 (Illumina).

Using a customized analysis pipeline (Figure S1B) all samples were run together on a virtual machine. The analysis pipeline implemented as a bash shell script is composed of TopHat^34^, Picard, (http://picard.sourceforge.net/index.shtml), Samtools^35^ and Cuffdiff^34^. TopHat aligns RNA-seq reads to the reference genome (GRCh37). Picard removes PCR duplicates and Samtools creates indices for bam files. We ran the Cuffdiff function from the Cufflinks package v2.1.1.^34^ to find differentially expressed genes and transcripts in the samples using the default settings. Results from Cuffdiff were plotted in R using the Bioconductor CummeRbund package^34^.

Information for mapped, unmapped and duplicate reads was extracted using Samtools. The length of the transcript in base pairs was calculated by summing up depth per genome position. The covered fraction of transcript was computed by dividing the total number of bases in mapped reads with the length of the transcript. Depth per genome position was calculated using GenomeCov function from BedTools^36^. RNA quality control was performed with RNA-SeQC^37^. Read metrics are summarized in Table S1B.

### Sample preparation for mass spectrometry

Subcellular fractionation was performed using the Nuclear Extract Kit (ActiveMotif) resulting in cytosolic (CYT), nuclear (NUC) and chromatin-bound (CH) proteins. All extraction buffers were supplemented with protease inhibitors (Complete EDTA-free, Roche) and phosphatase inhibitors (PhosStop, Roche). Protein was methanol/chloroform precipitated^38^ and resuspended in urea/thiourea buffer (6 M/2 M, 30 mM HEPES, pH 8). Protein concentration was determined by Bradford. Unlabeled and SILAC (M, H) samples were mixed at a ratio 1:1:1 using 60 μg of protein per sample. After reduction (5 mM DTT, 30 min) and alkylation (10 mM iodoacetamide, 20 min, in the dark), proteins were digested for 3 h by Lys-C (Wako) at a protein:enzyme ratio of 100:1. Samples were diluted with 10 mM ammonium bicarbonate to 2 M urea prior to overnight trypsin digestion (Sequencing grade, Promega) at a protein:enzyme ratio of 100:1.

### Isoelectric focusing

Peptides (180 μg) were separated by in-solution isoelectric focusing (OFFGEL fractionator, Agilent) into 12 fractions over a pH range of 3–10. Fractionation was performed according to manufacturer’s protocol with adaptation. Glycerol in the running buffer was reduced to 0.3% (original: 6%) and the ampholytes to 0.1% (original: 1%). Peptides were focused for 20 kVh. Peptides were harvested and wells incubated with 50 μl 50:49:1 methanol:H_2_O:TFA for 15 min, pooling the wash with the corresponding fractions. Fractionated peptides were dried down with a speedvac concentrator and kept at −80 °C until further use. Peptides were solubilized by 1% acetonitrile/0.05% TFA and desalted on C_18_ STAGETips^39^. Briefly, STAGETips were packed with 3 C_18_ disks and activated with methanol. After 2 washes of 2% acetonitrile/0.1% TFA, peptides were loaded. STAGETips were washed with 0.1 % acetic acid and 2% acetonitrile/0.1% TFA before elution with 60% acetonitrile/0.1% TFA. Eluates were dried down completely in a speedvac concentrator and reconstituted in 10 μl of mobile phase A (0.5% acetic acid) prior LC-MS/MS analysis.

### Mass spectrometry

#### Nano LC-MS/MS analysis

Of the resolubilized peptide solution, 6 10 μl were subsequently analyzed by liquid chromatography (LC) using an EASY nLC-II coupled to a Q Exactive mass spectrometer (Thermo Scientific, Bremen, Germany)^33^. Emitter columns were packed in-house with ReproSil-Pur 120 C18-AQ 3 μm diameter beads (Dr. Maisch GmbH, Germany). Liquid chromatography analysis was performed with a segmented gradient from 10 to 60% mobile phase B over 100 min at a flow rate of 250 nl/min. Mobile phase composition was as follows: mobile phase A, H_2_O with 0.5% acetic acid; mobile phase B, H_2_O: acetonitrile (20:80 volume ratio with 0.5% acetic acid). A data dependent mass spectrometric method was employed, where the 10 most intense ions, excluding unassigned charge states and singly charged ions, detected in the preceding full scan are isolated (3 Th isolation width) and fragmented using higher energy collisional dissociation (HCD) (normalized collision energy 25). Precursor scans (MS^1^ level) were acquired at a resolution of 70,000 (*m/z* 300) and an AGC target value of 3,000,000 charges (maximum ion injection time 20 ms). Fragmentation spectra were acquired at a resolution of 17,500 (m/z 300) and an AGC target value of 100,000 charges (maximum ion injection time 120 ms). All scan events were recorded in profile mode. A dynamic exclusion list of 25 s was employed and the exclude isotopes functionality was activated.

#### Data analysis

MS data was analyzed by MaxQuant suite of algorithms version 1.4.1.2^40^ using a Homo Sapiens database downloaded from UniprotKB on the 27^th^ of November 2013 and comprising 88,473 protein isoforms entries. The following default search parameters were employed: first search mass accuracy tolerance 20 ppm, main search mass accuracy tolerance 4.5 ppm, FTMS MS/MS tolerance 20 ppm, minimum peptide length of 7 amino acids, peptide spectrum match FDR and protein FDR both set to 0.01 as calculated by the revert database approach^41^. Protein quantification was based on a minimum of two ratio counts, originating from unique or razor peptides only. Additionally, unless explicitly stated otherwise, other parameters of the data analysis were not changed from their MaxQuant 1.4.1.2 default value.

Multiplicity was set to 3 with Arg6/Lys4 and Arg10/Lys8 for medium and heavy labels respectively as required by the triple SILAC approach and the search was conducted using Trypsin/P enzyme specificity, allowing for maximum two missed cleavages, N-terminal acetylation and methionine oxidation as variable modifications and cysteine carbamidomethylation as fixed modification. Both re-quantify and match between runs (match time: 1 min, alignment time window: 20 min) functionalities were enabled. MaxQuant analysis was performed both on the combined (36 MS runs per sample) and separate organelle fractions (12 MS runs per sample). Despite reducing the ampholyte concentration during in-solution isoelectric focusing (0.1% instead of the recommended 1%) and additional washing on C_18_ STAGETips, ampholytes eluted in the beginning of the chromatogram, interfering with timely MaxQuant processing. Raw files were thus preprocessed with the RecalOffline application (ThermoFisher Scientific) to exclude the first 35 min of acquisition.

#### Bioinformatics and statistical analysis

Proteomics data sets were analyzed with the empirical Bayes moderated T test implemented by the limma bioconductor package in the R environment. P values were corrected for multiple hypothesis testing using the Benjamini-Hochberg method (FDR ≤ 0.05). Differential expression was calculated on normalized log_2_ ratios (nano LC-MS/MS). Differential expression for RNA-sequencing data was performed with the Cufflinks package^34^ using the Cuffdiff function. PCA analysis was performed with the FactoMineR package v.1.29 within the R environment on FPKM values for RNA and on non-normalized intensities derived from mass spectrometry for PROT. Differentially expressed genes and proteins were processed using the in-house developed Comics R package for enrichment analysis.

#### Data availability

The mass spectrometry proteomics data, including all raw files and data analysis tables produced by MaxQuant analysis, have been deposited to the ProteomeXchange Consortium (http://proteomecentral.proteomexchange.org) via the PRIDE partner repository^42^ with the dataset identifier PXD001856. RNA sequencing data can be accessed via the Sequence Reads Archive by NCBI (BioProject ID: PRJNA277616).

## Results and Discussion

### Deep transcriptome analysis by RNA sequencing

As a first step in the comprehensive characterization of mesenchymal stem cells (ESC and bone marrow derived) we comparatively analyzed the transcriptome by deep sequencing. Currently, a single study is published characterizing ESC-MSC with deep RNA sequencing. The cells used, however, were derived by co-culture with OP9 cells^43^ and are thus not compatible with clinical usage.

**Figure 1:**
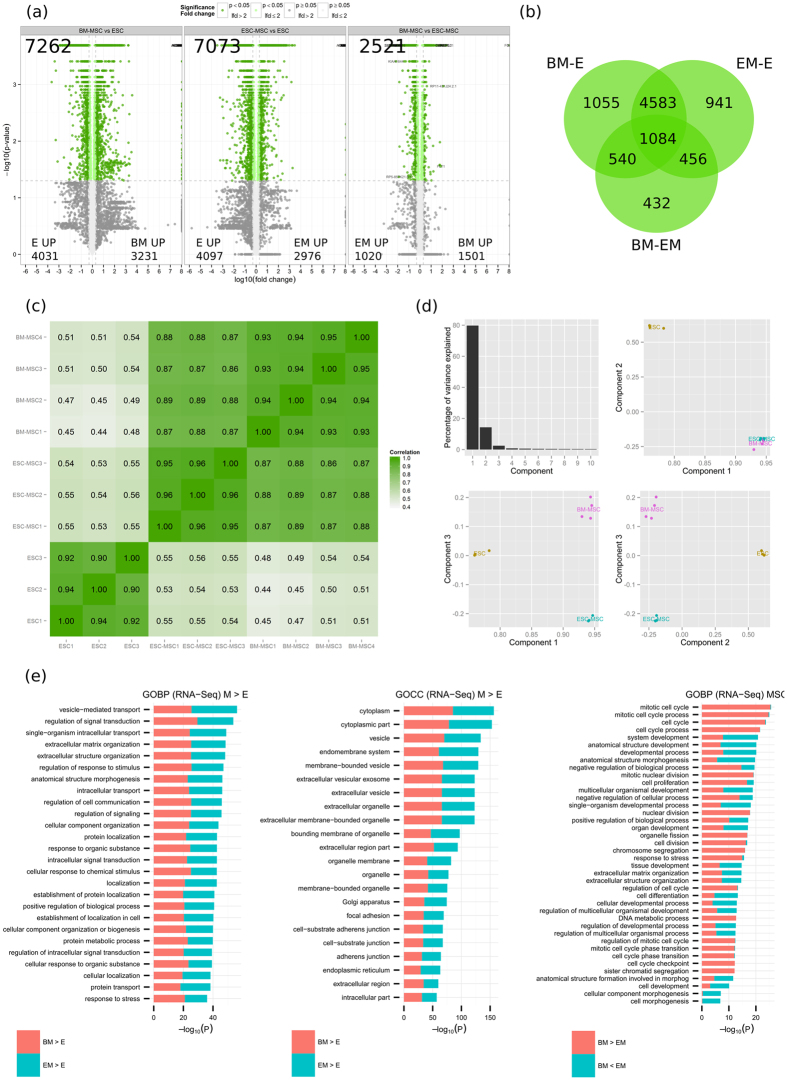
Transcriptome analysis (RNA) of ESC-MSC (EM), BM-MSC (BM) and ESC (E) by RNA-seq. (**a**) Volcano plots for BM-MSC vs ESC (left panel), ESC-MSC vs ESC (middle panel) and BM-MSC vs ESC-MSC (right panel). Numbers are given for the total of differentially expressed genes as well as for the up-regulated per cell type. (**b**) Venn diagram of SDEs per comparisons (BM-MSC vs ESC, ESC-MSC vs ESC and BM-MSC vs ESC-MSC). (**c**) Pearson correlation matrix for all replicates. (**d**) PCA analysis based on FPKM values; principal components 1 to 3 were plotted against each other. (**e**) GO-term enrichment analysis for biological process (GOBP) and cellular component (GOCC). Bar charts represent the most significant top 20 terms of each category for each cell type sorted by mean –log_10_ p values.

The RNA-seq pipeline is depicted in Figure S1B. Differential expression analysis by CuffDiff^34^ led to 7262, 7073 and 2521 modulated genes (FDR ≤ 0.05) for BM-MSC vs ESC (BM-E), ESC-MSC vs ESC (EM-E), and BM-MSC vs ESC-MSC (BM-EM), respectively. Among those are 3578, 3394 and 1001 modulated isoforms (FDR ≤ 0.05) corresponding to 2452, 2285 and 868 genes for BM-E, EM-E and BM-EM (Fig. 1; Figure S2A,B; Table S1). As a control, a set of pluripotency and MSC markers were analyzed and as expected, separation of the cell types (ESC and MSC) was observed (Figure S2C). A Pearson correlation matrix clearly demonstrates the differences between ESC and MSC, but also shows that the two MSC subpopulations (ESC-MSC and BM-MSC) segregate. ESC-MSC are closer to ESC (0.53–0.56) than BM-MSC to ESC (0.44–0.54). The two younger BM-MSC donors (27y, 20y – BM-MSC 3 and 4 respectively) are closer to ESC (0.5–0.54) than the other two (40y, 39y– BM-MSC 1 and 2 respectively) (0.44–0.49). This may be interpreted as ESC-MSC representing a particularly young subclass of MSC. Correlations between both MSC populations range from 0.86–0.89, whereas intra-group correlations range from 0.95–0.96 (EM) and 0.93–0.95 (BM) (Fig. 1c). ESC to MSC differences may be explained in PCA analysis by the first two components covering 94% of variance. Component 3 separates the two MSC populations, explaining less than 5% of variance. As in the correlation matrix, BM-MSC donors show a larger intra-group variation than their ESC-MSC counterparts (Fig. 1d).

The observed differences between the two MSC populations are similar in scope to the variability recently observed between differentially sourced MSC^44^,^45^. They may thus be reflective of ESC-MSC representing yet another sourcing-equivalent MSC subtype or indicate that ESC-MSC are closer to adult MSC from a different source, sharing expression differences and differentiation potential. This hypothesis is further strengthened in a direct side-by-side comparison with a published data set covering fetal (placenta-derived) and adult (bone marrow-derived) MSC^45^ using our bioinformatics pipeline. A similar distance between the two MSC subtypes was observed (Figure S3).

When comparing up-regulated genes in MSC versus ESC (BM > E, EM > E), only minor changes can be observed between the two MSC subpopulations. Top enriched biological processes (GOBP) in MSC are vesicle-mediated transport, regulation of signal transduction and ECM organization. Enriched terms of the cellular compartment category (GOCC) include cytoplasm, vesicle, exosomes, organelle membrane, focal adhesion and Golgi apparatus. Tissue regeneration initiated by MSC is known to happen in a paracrine manner and the observed strong enrichment of exosomes as well as vesicle-mediated transport further confirms the central role of this process in regeneration support. Notably, developmental processes with a wide range of different tissues are enriched in MSC (Figure S6A, left, Table S3). Those include blood vessels, neurons, skeleton, muscle, heart, connective tissue, cartilage, kidney, gland, epithelium, urogenital tract, eye and others, reflecting the wide range of MSC application in clinical and laboratory research.

MSC from different sources, although expressing the classical surface markers (CD73, CD90, CD105) and exhibiting similar trilineage differentiation potential, show distinct molecular signatures, as previously reported^44^,^45^,^46^. Most significant changes between both MSC populations were observed for membrane-bound glycosylated or secreted proteins, indicating differential signaling or homing properties. Functionally, those proteins from both MSC populations are enriched for ECM organization, cell differentiation, and developmental processes (Fig. 1e, Figure S6A). Uniquely enriched among up-regulated genes of BM-MSC are genes associated with mitotic cell cycle and regulation of proliferation, which is counterintuitive since BM-MSC are slower in their proliferation rate than ESC-MSC. However, upon applying a fold change cutoff of 2 log_2_, all cell cycle related functions disappear, indicating the relative low amplitude of the change, possibly originating from overall low signal to noise of the associated probes. Transcripts present exclusively in BM-MSC (n = 71) are enriched in extracellular vesicle proteins. Among the RNA transcripts present only in ESC-MSC (n = 19) are four transcription factors or regulators involved in developmental processes (HOXD1, NKX2–5, LHX2, FGF12), concordant with our previous study where we described the increased differentiation ability of these MSC^47^. The majority of ESC-MSC specific transcripts (n = 12) is unknown, assigned to neither genes nor noncoding RNAs.

### LC-MS/MS-based proteomics combined with SILAC

For the quantitative proteomic analysis, experiments were designed to use triple SILAC labeling with ESC, ESC-MSC and BM-MSC labeled as “light”, “medium” and “heavy”, respectively. Three biological replicates were generated including label swapping. Biological triplicates for BM-MSC originated from three donors, whereas ESC-MSC triplicates were derived from independent differentiation experiments. For increased proteome coverage, subcellular fractionation into cytosol (CYT), nucleus (NUC), and chromatin (CH) was performed^48^ (Figure S4A,B,C). Three markers have been selected to verify the enrichment of cytosolic, nuclear and chromatin-bound proteins by westernblot (Figure S4D). To validate the fractionation, the dual specificity mitogen-activated protein kinase kinase 2 (gene: MAP2K2) was selected as a representative cytosolic protein, histone H2AZ (gene: H2AFV) as exclusively chromatin-bound, and lysine-specific histone demethylase 1 (gene: KDM1A) as present in both nuclear and chromatin-bound fractions. All three proteins were measured by mass spectrometry and their intensity profiles (Figure S4E) validated the western blot data and confirmed the specificity of the organelle fractionation technique employed. In the combined analysis (CYT, NUC and CH together), 8408 proteins were identified with 7129, 7023 and 7136 quantified proteins per replicate (FDR ≤ 1% on both peptide and protein level) (Figure S4F, Table S2). As an internal control, the data set was screened for proteins expected in ESC and MSC, respectively. Pluripotency-associated proteins identified include SOX2, POU5F1, SALL4, UTF1, DNMT3B, DPPA4, LIN28A, and GRB7. As classical MSC phenotyping surface markers^49^ we identified ALCAM (CD116), ITGB1 (CD29), CD44, ENG (CD105), NT5E (CD73), and THY1 (CD90) (Figure S4G). As expected, expression patterns observed followed the cell type origin. For instance, bone marrow stromal cell antigen 1 (BST1, CD157), found to be only enhanced in BM-MSC, is a MSC marker involved in self-renewal, migration and osteogenic differentiation^50^.

**Figure 2:**
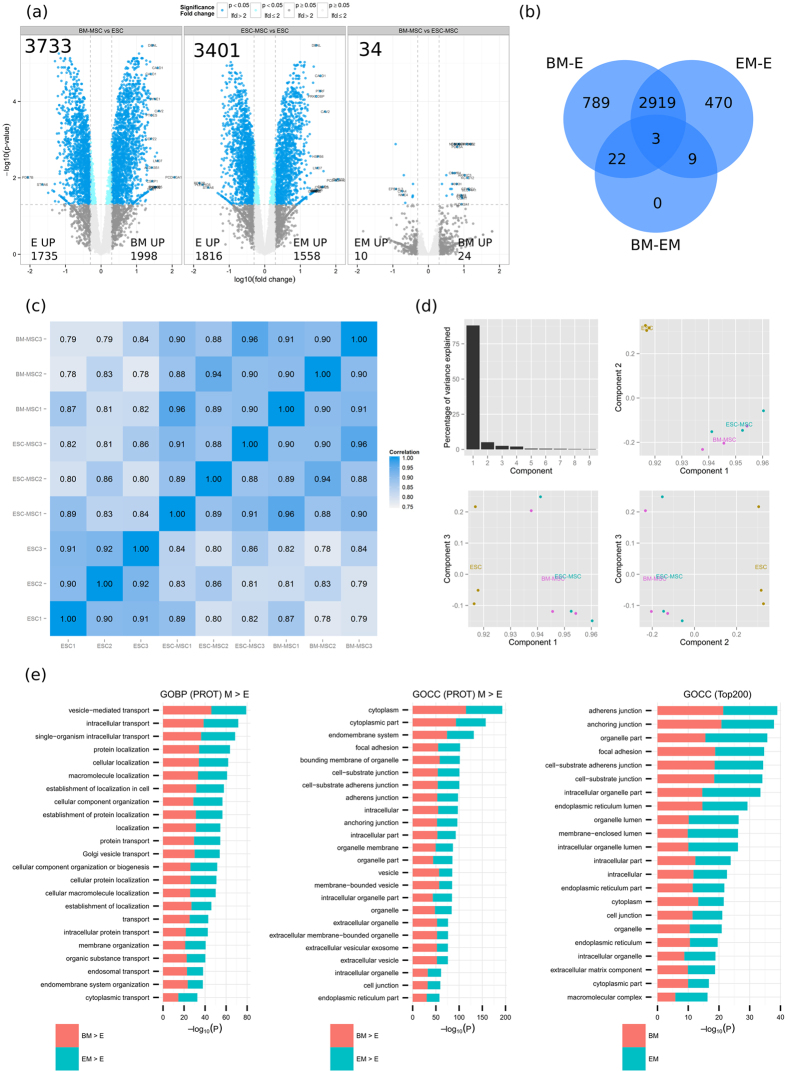
Proteome analysis (PROT) of ESC-MSC (EM), BM-MSC (BM) and ESC (E) by nano LC-MS/MS. (**a**) Volcano plots for BM-MSC vs ESC, ESC-MSC vs ESC and BM-MSC vs ESC-MSC. Numbers are given for the total of differentially expressed proteins as well as for the up-regulated per cell type. (**b**) Venn diagram of SDEs per comparisons (BM-MSC vs ESC, ESC-MSC vs ESC and BM-MSC vs ESC-MSC). (**c**) Pearson correlation matrix for all replicates. (**d**) PCA analysis based on non-normalized log_10_ intensities was performed; principal components 1 to 3 were plotted against each other. (**e**) GO-term enrichment analysis for biological process (GOBP) and cellular component (GOCC). Bar charts represent the most significant top 20 terms of each category for each cell type sorted by mean –log_10_ p values.

Differential expression of the combined analysis was assessed by the empirical Bayes moderate T test (limma, FDR ≤ 0.05) with 3733, 3401 and 34 proteins differentially expressed between BM-E, EM-E and BM-EM, respectively (Fig. 2a,b). Unlike RNA sequencing, only a low number of proteins are differentially expressed between BM-MSC and ESC-MSC. When reporting correlation coefficients for all samples based on their non-normalized log_10_ raw intensities across all experiments (Fig. 2c) ESC and MSC clearly separate. Correlation coefficients between ESC and MSC range from 0.86–0.89 for ESC-MSC and 0.83–0.87 for BM-MSC, while the two MSC subtypes correlate in the range 0.94–0.96. The close relationship between ESC-MSC and BM-MSC is also validated by PCA analysis, where approximately 95% of the variance is explained by the first two components separating ESC and MSC (Fig. 2d).

Comparing our proteomic dataset on ESC and MSC to the two most comprehensive human proteome maps published to date, which contain together 19608 proteins and cover a wide range of tissues from adult and fetal origin^51^,^52^, we uniquely identified 54 proteins and 5409 peptides, which map to 3024 proteins (Figure S5A). Our study thus provides new additional data to the human proteome. Given their likely stem cell specificity and the origin of the data in the maps, the 54 proteins are unsurprisingly largely uncharacterized and thus lack functional data.

In comparison to the most comprehensive studies available on human ESC^53^,^54^ (Figure S5B), our data set contains 4500 proteins commonly expressed within the three studies which may be seen as an ESC core proteome. The data set adds 1661 unique identifications, expanding the ESC proteome and contributing to ESC biology. Finally, comparing our data set to the most comprehensive MSC proteomics study available to date^55^, our relative contribution to the reported MSC proteome is even larger adding 3331 unique identifications (Figure S5C).

Among all proteins identified, 62 were classified as uncharacterized proteins including KIAAs and ORFs, from which 20 were significantly differentially expressed, hinting at a role in stem cell biology. A large proportion of transcription-related proteins in our dataset are zinc finger proteins (ZNF) (207) which is believed to be the largest family of transcription factors in mammalians. Most of these ZNF remain uncharacterized^56^, but some have been shown to be implicated in developmental processes^57^,^58^, with recent examples involving chondrogenesis^59^ (ZNF449) and adipogenesis^60^ (ZNF395). Therefore, the differentially expressed ZNF proteins observed here in ESC and MSC strongly suggests they also play an important role in stem cell biology. The highest up-regulated protein in ESC-MSC is ZNF322 (177 fold), associated with MAPK signaling and heart development^61^. ZFHX4, up-regulated in both ESC-MSC and BM-MSC is involved in neuronal differentiation^62^. FHL2, up-regulated in both MSC populations as well, controls osteogenic differentiation^63^. Based on bioinformatics analysis we observed an enrichment of zinc fingers of the LIM-type in both MSC types. Functionally, these are associated with cytoskeleton organization and focal adhesion, but also tissue-specific differentiation^64^.

The human proteome map published by the Pandey group^51^ includes tissues and cell types of fetal and adult origin. Among the 30 tissues analyzed, 2350 proteins were found to be expressed ubiquitously and can be considered house keeping proteins or core proteome. Filtering those out of protein lists should thus facilitate the selection of signature proteins that define cell type functionality. GO term enrichment analysis was thus performed on the 200 most intense proteins of ESC-MSC and BM-MSC after removing the core proteome (Fig. 2e). The top biological processes enriched in the 200 most intense MSC proteins include extracellular matrix organization, adhesion and developmental processes (vasculature and endoderm). As seen with RNA-seq, vesicle-mediated transport, focal adhesion and vesicles/exosomes are among the most enriched terms in MSC when comparing to ESC (Fig. 2e).

### Data integration of the two large-scale techniques

**Figure 3:**
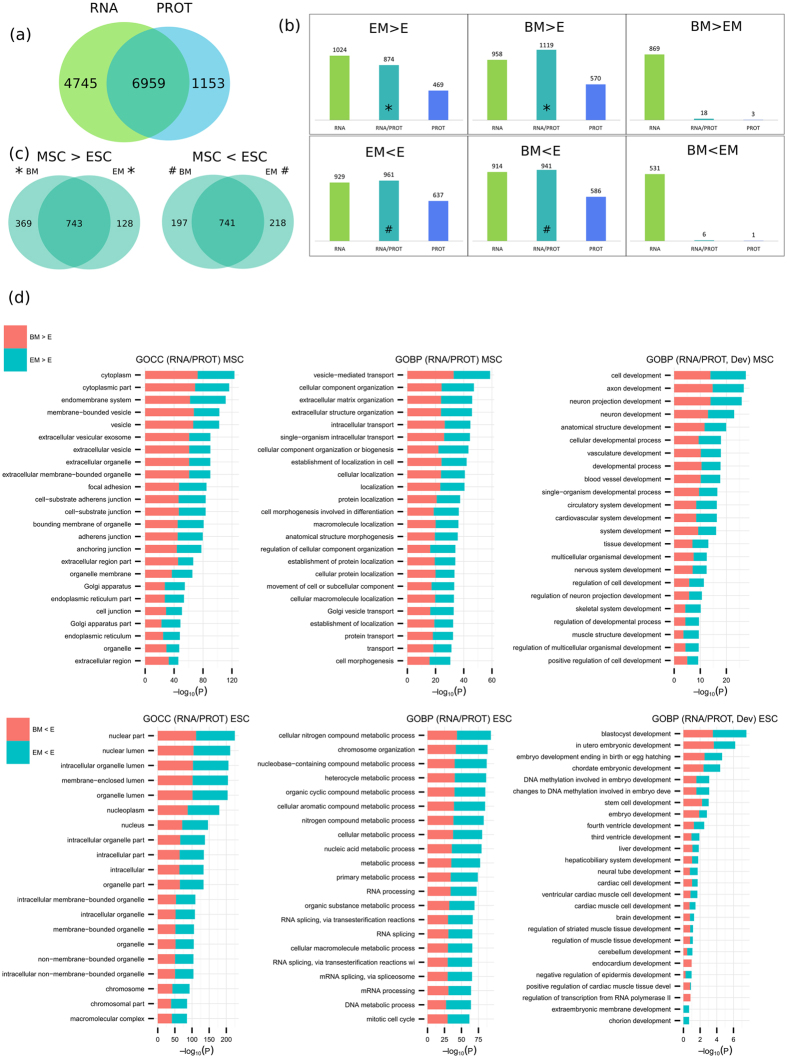
Combined analysis (RNA, PROT) of MSC and ESC. (**a**) Comparison of identified features based on gene name. (**b**) Comparison between significant differentially expressed proteins (SDEs) (FDR ≤ 0.05) found by RNA and PROT for all six cases: EM > E, BM > E, BM > EM, EM < E, BM < E, BM < EM; comparisons are based on common genes/proteins identified by RNA and PROT. (**c**) Venn diagrams display SDEs of genes/proteins up-regulated in MSC (left) and ESC (right) and validated by both techniques (RNA and PROT). (**d**) GO-term enrichment analysis on RNA and PROT validated genes/proteins up-regulated in MSC (top) and ESC (bottom). Bar charts represent the most significant top 20 terms of each category per cell type and sorted by mean −log_10_ p values. In addition to GOCC (left panels) and GOBP (middle panels), an enrichment analysis for GOBP development terms is presented (right panels).

A large overlap of identifications can be obtained (6959) when comparing RNA-seq and LC-MS/MS data (Fig. 3a), extending to those measured as differentially expressed. 60% of proteomic (LC-MS/MS) changes were validated by RNA-seq (Fig. 3b). Both techniques give very similar results in terms of global trends of expression as emphasized by the Pearson correlation coefficient of 0.66 in agreement with the literature^65^.

### Fundamental differences between ESC and MSC

ESC and MSC differ extensively as demonstrated by PCA analysis for both techniques (Figs 1d, 2d). RNA-seq and proteomics validated genes/proteins differentially expressed between ESC and MSC (Fig. 3c) were subjected to enrichment analysis (Fig. 3d). Upon performing the analysis on cellular component associated terms (GOCC): cytoplasm, endomembrane system, vesicle, extracellular exosomes, focal adhesion, and Golgi apparatus were associated to proteins/genes up-regulation in MSC. Consistently, the biological processes (GOBP) found enriched in MSC by both techniques included vesicle-mediated transport, cellular component organization, extracellular matrix organization and intracellular transport. When refining the GOBP analysis by filtering for developmental terms, the top 20 enriched processes in MSC included: axon, neuron projection, neuron, vasculature, cardiovascular system, skeletal and muscle development. In total, 45 developmental terms (p val ≤ 0.05) were commonly enriched for both MSC subtypes (ESC-MSC and BM-MSC) by RNA and PROT, including for instance, and in addition to the above mentioned, heart, cartilage, mesenchyme, bone, and endoderm development. If considering developmental terms found exclusively by RNA-seq (p val ≤ 0.05), then kidney, gland, liver, eye, and urogenital system development may be added to the list.

Comparatively, upon conducting the analysis on cellular component associated terms (GOCC) for proteins/genes up-regulated in ESC, nucleus, nuclear lumen, nucleoplasm, nucleolus, and chromosome showed significant enrichment. The corresponding top biological processes included chromosome organization, RNA processing, RNA splicing and cell cycle. These findings reflect the highly proliferative nature of ESC (cell division time of 15–16 h as compared to 2–3 d in MSC^66^) and high relative contribution of the nucleus to total cell volume in this cell type.

### How do ESC-MSC compare to BM-MSC

Central to answering the question on usability of ESC-MSC in clinical applications is their quantitative comparison to the currently employed standard: BM-MSC. RNA-sequencing reported a total of 2521 characteristic features differentially expressed between the two MSC populations, with 1501 and 1020 respectively up- and down-regulated when comparing BM-MSC to ESC-MSC (Fig. 1a). However, LC-MS/MS based analysis reported minimal differences with only 34 significantly differentially expressed proteins, 24 up- and 10 down-regulated, when comparing BM-MSC to ESC-MSC (Fig. 2a). This is presumably due to a lower analytical depth of PROT compared to RNA as well as an increased stringency of the MS-based statistical analysis, but also reflects changes at the RNA level that did not systematically translate to the protein level (Fig. 3b).

**Figure 4:**
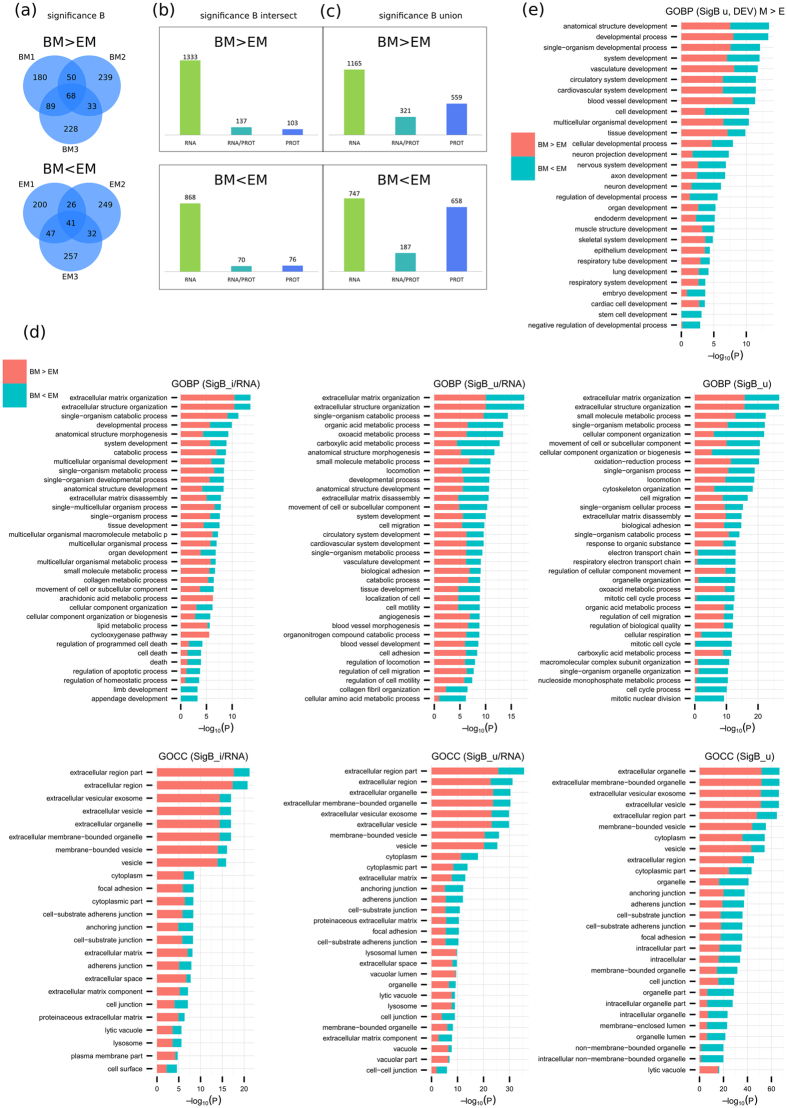
Comparison of BM and EM based on significance B. (**a**) Venn diagrams display PROT SDEs for BM > EM (top) and BM < EM (bottom) by significance B (p value ≤ 0.05) per replicate. Comparison to RNA results based on (**b**) intersecting proteins or (**c**) the union. (**d**) Enrichment analysis on BM vs EM based on significance B analysis of RNA validated candidates for proteins measured by PROT in at least 2 replicates (SigB_i/RNA, left) or 1 replicate (SigB_u/RNA, middle) as well as all significance B SDE proteins measured by PROT (SigB_u, right). Bar charts represent the most significant top 20 terms of GOBP (top) and GOCC (bottom) per cell type and sorted by mean −log_10_ p values. (**e**) GOBP development terms specific enrichment analysis for GOBP terms enriched for significance B SDE proteins measured by PROT.

Applying significance B statistical analysis^40^ instead of limma allowed for a more extensive comparison between PROT and RNA-seq results. A total of 1739 significantly differentially expressed proteins, 887 overexpressed in BM-MSC and 852 overexpressed in ESC-MSC, were reported using this approach (Fig. 4a). SDE proteins in at least two replicates (Fig. 4a intersections, Fig. 4b) and in any replicate (Fig. 4a union, Fig. 4c) were compared to RNA-seq data. Based on all intersecting significance B SDE proteins (240 for BM-MSC, 146 for ESC-MSC): 137 and 70 candidates (for BM-MSC and ESC-MSC respectively) were validated by RNA-seq. When considering the union of all significance B SDE proteins instead (887 for BM-MSC, 852 for ESC-MSC) those numbers increase to 321 and 187 validated proteins for BM-MSC and ESC-MSC, respectively.

GO term enrichment analyzes were performed for validated proteins (RNA/PROT overlap) of significance B intersection (Fig. 4d, left panels) and union (Fig. 4d, middle panels) as well as for all significance B SDE proteins measured by LC-MS/MS (Fig. 4d, right panels). Based on RNA validated intersecting significance B proteins, ECM organization and extracellular exosome pathways were found to be enriched in MSC. Upon decreasing stringency by including RNA validated proteins from the significance B union, similar functions were enriched at low p values, specifically developmental processes and exosomes. Furthermore, when considering all significance B SDE proteins identified in PROT only, a remarkably high enrichment of extracellular vesicle exosome (ESC-MSC: 212, p val 9.92E–16; BM-MSC: 307, p val 5.80E–52), ECM organization (ESC-MSC: 53, p val 1.13E–11; BM-MSC: 63, p val 1.71E–16) and developmental process (ESC-MSC: 320, p val 4.31E-06; BM-MSC: 348, p val 9.55E–09) was observed. These results consistently point towards exosome production playing a central role in both MSC subpopulations, and overall close resemblance of both MSC subtypes. A finer grained comparison between ESC-MSC and BM-MSC highlights biological processes expression levels differences relating to their source: as expected cell cycle and nuclear division related processes are enriched in ESC-MSC, consistent with their higher proliferation rate, whereas BM-MSC show enhanced extracellular organization related processes, consistent with previously reported findings.

Filtering the biological process enrichment for developmental terms, as done for the ESC to MSC comparison, hints at other specificities distinguishing both MSC subtypes (Fig. 4e). Interestingly, ESC-MSC show higher enrichment for neuron and axon development whereas BM-MSC are more enriched for vasculature development. Additionally and as expected, ESC-MSC are also exclusively enriched in stem cell and embryo development terms. Overall, developmental terms related functions appear very similar in both MSC populations.

### Surface markers: CDs and other plasma membrane proteins

**Figure 5:**
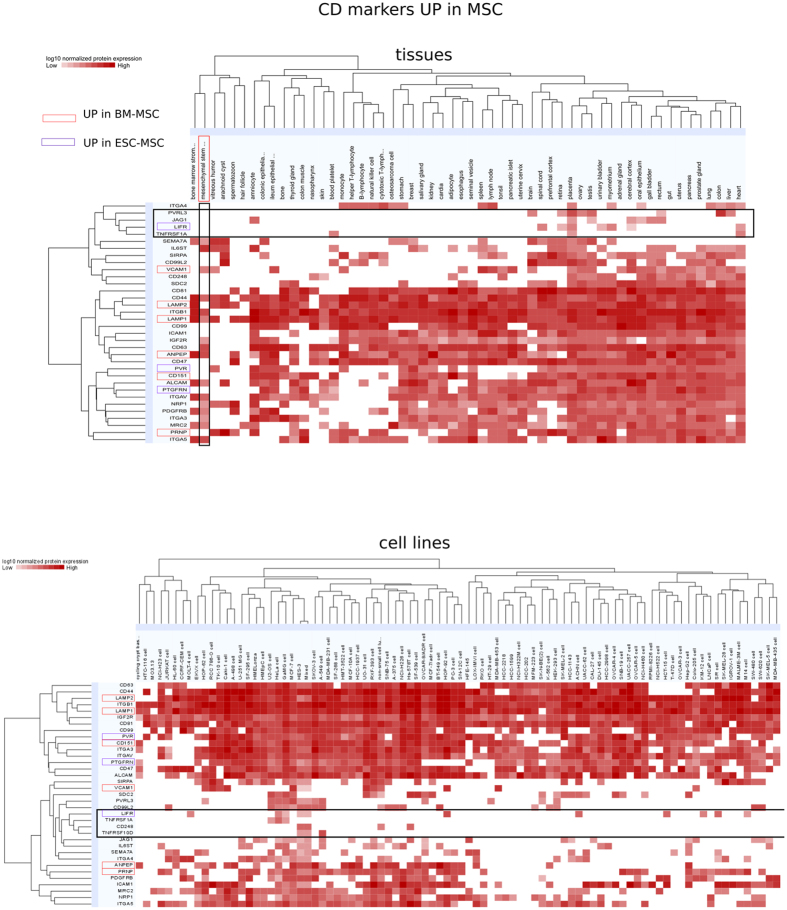
New potential MSC markers among CD molecules. 37 CD markers were found to be up-regulated in MSC when compared to ESC by RNA, PROT or both (Table S3). Their normalized log_10_ protein expression levels are shown across a wide range of tissues (top panel) and cell lines (bottom panel) according to ProteomicsDB. CD molecules up-regulated solely in ESC-MSC are highlighted in purple, whereas those up-regulated only in BM-MSC are highlighted in red. CD molecules potentially best suited as MSC markers are comprised in the horizontal rectangle frame.

Previously, several studies were conducted to catalogue MSC surface markers^67^,^68^, the most comprehensive to date including 116 CD markers found on bone marrow derived MSC^67^. Using a combination of RNA-seq and quantitative proteomics, we identified 137 surface markers of the CD family (Table S4). As expected, common MSC markers (CD29, CD36, CD44, CD73, CD90, CD105, CD106, CD166) were highly expressed, whereas hematopoietic markers (CD19, CD20, CD34, CD45) were absent. High expression levels of THY-1 (CD90), VCAM1 (CD106), and LNGFR (CD271) on MSC have been associated with self-renewal and robust lineage differentiation^69^ and were proposed as a quality control for clinical use of MSC. Among the SDE proteins validated by both techniques, 9 CD markers were found up-regulated in ESC (Table S4), and 28 CD markers were found up-regulated in both MSC subtypes; 3 and 6 specifically for ESC-MSC (LIFR, PTGFRN, PVR) and BM-MSC (VCAM1, CD151, LAMP1, ANPEP, PRNP, LAMP2), respectively (Fig. 5, Table S4). Among the 28 CD markers enriched in MSC were several tetraspanins known to be exosome markers such as CD9P, CD63, CD81, and CD151^70^. Protein expression profiles of aforementioned MSC CD markers are shown for a broad range of tissues and cell lines accessible through ProteomicsDB^52^ (Fig 5). The potentially best suited MSC markers are TNFSF1A, LIFR, JAG1 and PVRL3, when considering tissues, or TNFRSF10D, TNFRSF1A, CD248 and LIFR, when considering cell lines. In general, most discriminative are TNFRSF1A (CD120a) and TNFRSF10D (CD264), and their use, or more generally our findings, may prove helpful in refining the definition of MSC as given by^67^,^68^.

**Figure 6:**
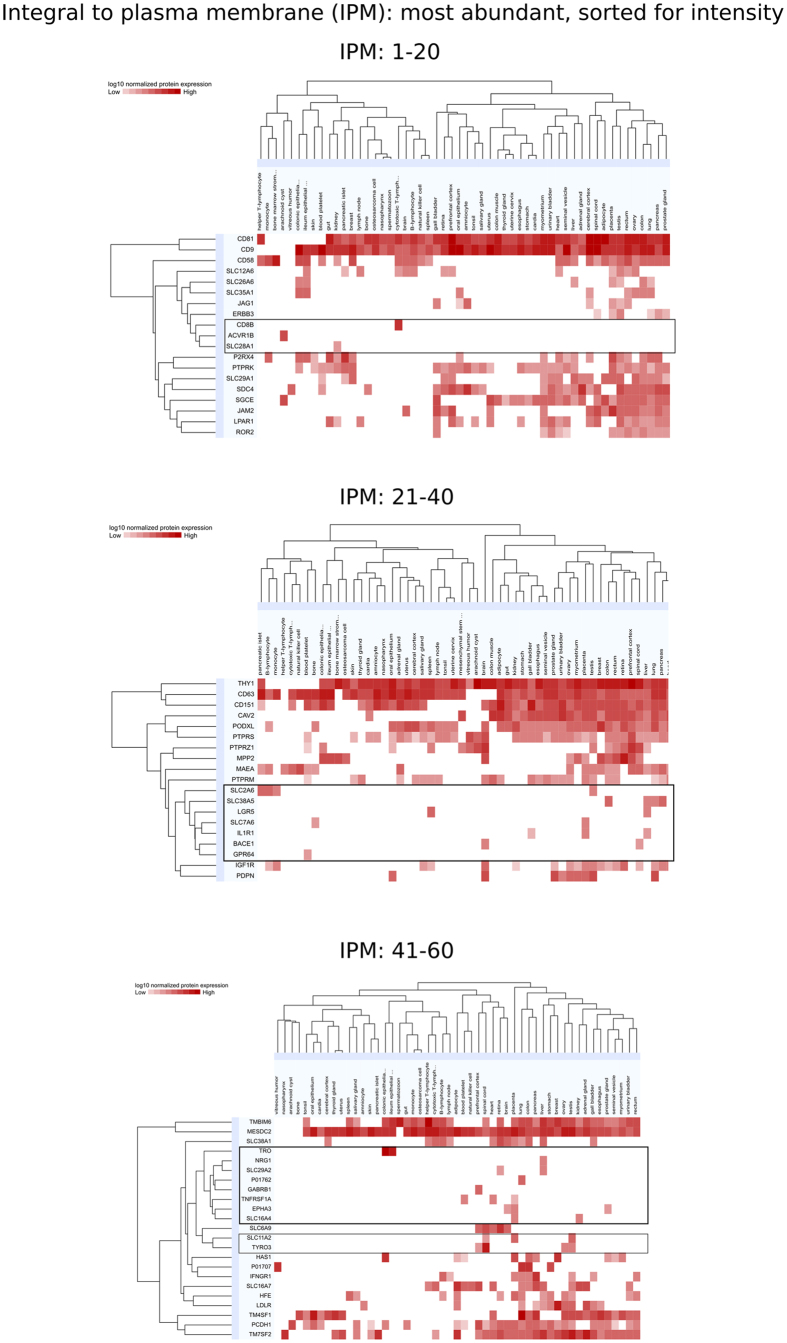
New potential MSC markers among plasma membrane proteins. Proteins identified in MSC (by proteomics) annotated as “integral to plasma membrane” (IPM) were screened for overall tissue expression based on ProteomicsDB. Heatmaps of the 60 most intense IPM proteins for EM and BM are shown, sorted by tissue type and normalized log_10_ protein expression and separated in 3 panels as follows: IPM proteins 1 to 20 (upper panel), 21 to 40 (middle panel) and 41 to 60 (lower panel). Potential MSC markers are comprised in the horizontal rectangle frames.

To broaden our search for potential MSC surface markers, our dataset was further filtered for proteins integral to plasma membrane; 293 candidates identified by nano LC-MS/MS in both MSC subtypes were thus obtained. Tissue expression profiles for the 60 most intense candidates among those membrane proteins were then generated using ProteiomicsDB^52^ (Fig. 6). Out of those 60 most intense plasma membrane proteins, 20 displayed tissue specificity, defined as detectable expression levels in a maximum of 4 out of 56 representative tissues. On the basis of this definition, those membrane proteins could be considered additional potential MSC markers and include: ACVR1B, BACE1, CD8B, EPHA3, GABRB1, GPR64, IL1R1, LGR5, NRG1, P01762, SLC2A6, SLC7A6, SLC11A2, SLC16A4, SLC28A1, SLC29A2, SLC38A5, TNFRSF1A, TRO, and TYRO3. Among the non-CD surface markers with high intensity and the most specific expression (one out of 56 tissues) were ACVR1B, GABRB1, GPR64, NGR1, P01762 and SLC28A1.

## Conclusions

Using a combination of RNA sequencing and quantitative MS-based proteomics, we report here the most comprehensive proteome to date for both embryonic and mesenchymal stem cells, quantifying transcripts deriving from more than 15000 genes and quantitatively profiling more than 8400 proteins.

Both techniques demonstrate high similarity of expression profiles for the two MSC subtypes and their marked difference to the ESC proteome. RNA sequencing allowed to identify approximately 2500 significantly differentially expressed (SDE) genes when comparing BM-MSC and ESC-MSC, while MS-based proteomics reported 40 to 200 SDE proteins between the MSC subtypes (depending on the statistical analysis stringency). It is worth noting that while the sensitivity advantage of nucleic acid based techniques may lead to overestimation of differences through emphasis of biological noise, true biological differences may also be masked in mass spectrometry by dynamic range limitations. The higher number of SDE features measured by RNA sequencing may also relate to translational and post-translational regulation not propagating into protein abundance differences. Nonetheless, a 60% correlation coefficient between the two techniques was achieved, validating literature reports and confirming the quality of the presented dataset.

Based on PCA and correlation analysis, the presented data suggests equivalency between bone marrow- and embryonic stem cell-derived MSC, with differences similar to previous reports on donor-derived MSC from different tissues. This interpretation is further supported by the observation that expression profiles of BM-MSC from younger donors correlate better to those of ESC derived MSC. The reported differences between ESC and MSC reflect the expected cell behavior: cell cycle centered for the former versus extracellular processes driven for the latter. Proteins related to vesicle-mediated transport and exosomes were found to be central in MSC biology, supporting the paracrine functionality reported by others. The striking enrichment in both MSC subtypes for developmental terms points to the versatility of MSC, as reflected by their wide range of usage in trials. Apparent from enrichment analysis, ESC-MSC may support more neurogenesis related processes, whereas BM-MSC more vasculature development, suggesting the potential use of differentially sourced MSC to address more specific medical conditions. Overall and keeping in mind that the results may be specific to the isolation and culturing conditions used,we believe these findings support future evaluation of the clinical use of ESC-derived MSC as a viable substitute for their bone marrow derived counterparts.

Finally, we reported a total of 137 surface markers of the CD family expressed in ESC and MSC identified jointly in RNA-seq and LC-MS/MS. Among those, 28 were found up-regulated in both MSC subtypes including exosome markers (CD9P, CD63, CD81, and CD151), further confirming the central importance of extracellular processes in MSC biology. Comparing protein expression profiles for the reported MSC CD markers across a variety of cell lines and tissues accessible through ProteomicsDB highlighted potential novel MSC markers: TNFSF1A, TNFRSF10D, LIFR, JAG1, PVRL3 and CD248. Among those, LIFR displays ESC-MSC specificity which may prove of use for quality control towards clinical application. Additionally, we have considered membrane associated proteins as potential cellular markers and have identified 20 tissue specific candidates from the 60 most intense plasma membrane proteins, among which ACVR1B, GABRB1, GPR64, NGR1, P01762 and SLC28A1 were the most promising. We believe MSC definition and, in particular, immunofluorescence-based applications may greatly benefit from the presented list of specific CD markers and membrane associated proteins.

## Additional Information

**How to cite this article**: Billing, A. M. *et al*. Comprehensive transcriptomic and proteomic characterization of human mesenchymal stem cells reveals source specific cellular markers. *Sci. Rep.* **6**, 21507; doi: 10.1038/srep21507 (2016).

## Supplementary Material

Supplementary Information

Supplementary Table S1

Supplementary Table S2

Supplementary Table S3
