# Supplementary material for: Comprehensive transcriptomic and proteomic characterization of human mesenchymal stem cells reveals source specific cellular markers
Source: Sci Rep. 2016 Feb 9;6:21507. doi: 10.1038/srep21507 (PMC4746666; doi:10.1038/srep21507)
Supplement: Supplementary Information [file srep21507-s1.pdf]

Supplementary Information

Comprehensive transcriptomic and proteomic characterization of human mesenchymal stem cells reveals source specific cellular markers

Anja M. Billing, Hisham Ben Hamidane, Shaima S. Dib, Richard J. Cotton, Aditya M. Bhagwat, Pankaj Kumar, Shahina Hayat, Noha A. Yousri, Neha Goswami, Karsten Suhre, Arash Rafii, Johannes Graumann*

Research Division, Weill Cornell Medicine-Qatar, Doha, State of Qatar

*corresponding author, Johannes Graumann, Ph.D., Assistant Professor of Biochemistry, Director, Proteomics Core, Weill Cornell Medicine – Qatar, Research Division, Qatar Foundation, Education City, P.O.Box 24144, Doha, State of Qatar, Tel.: +974 4492 8478 / Fax: +974 4492 8422, Skype: joh.graumann, Email: jog2030@qatar-med.cornell.edu

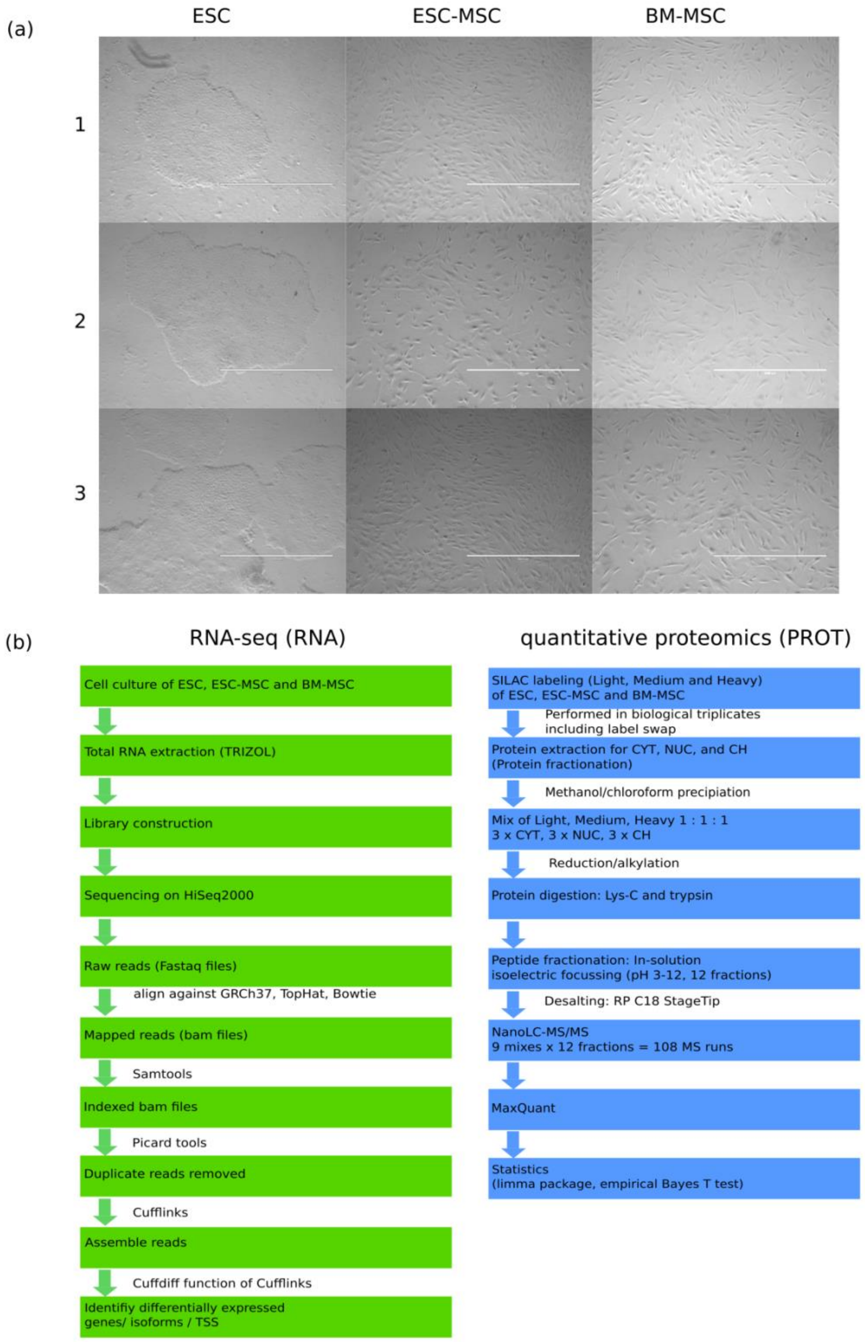

Supplementary Figure S1. **ESC-derived MSC (ESC-MSC) were compared to their origin ESC as well as to bone marrow-derived MSC (BM-MSC) with two high throughput techniques: RNA deep sequencing (RNA) and LC-MS/MS (PROT). (a)** Representative microscope images of ESC, ESC-MSC and BM-MSC for the three experiments. ESC-MSC in the study were derived from three independent differentiation experiments. BM-MSC were derived from four donors, with three of them used in proteomics. Details of the BM-MSC used; 40y/m (StemCell, MSC-001F, lot#BM2893), 39/m (Lonza, PT2505, lot#1F3422), 27y/m (Lonza, PT2505, lot#318006), 20y/m (only in RNA-seq, Lonza, PT2505, lot#8F3520). **(b**) Pipelines of RNA and PROT.

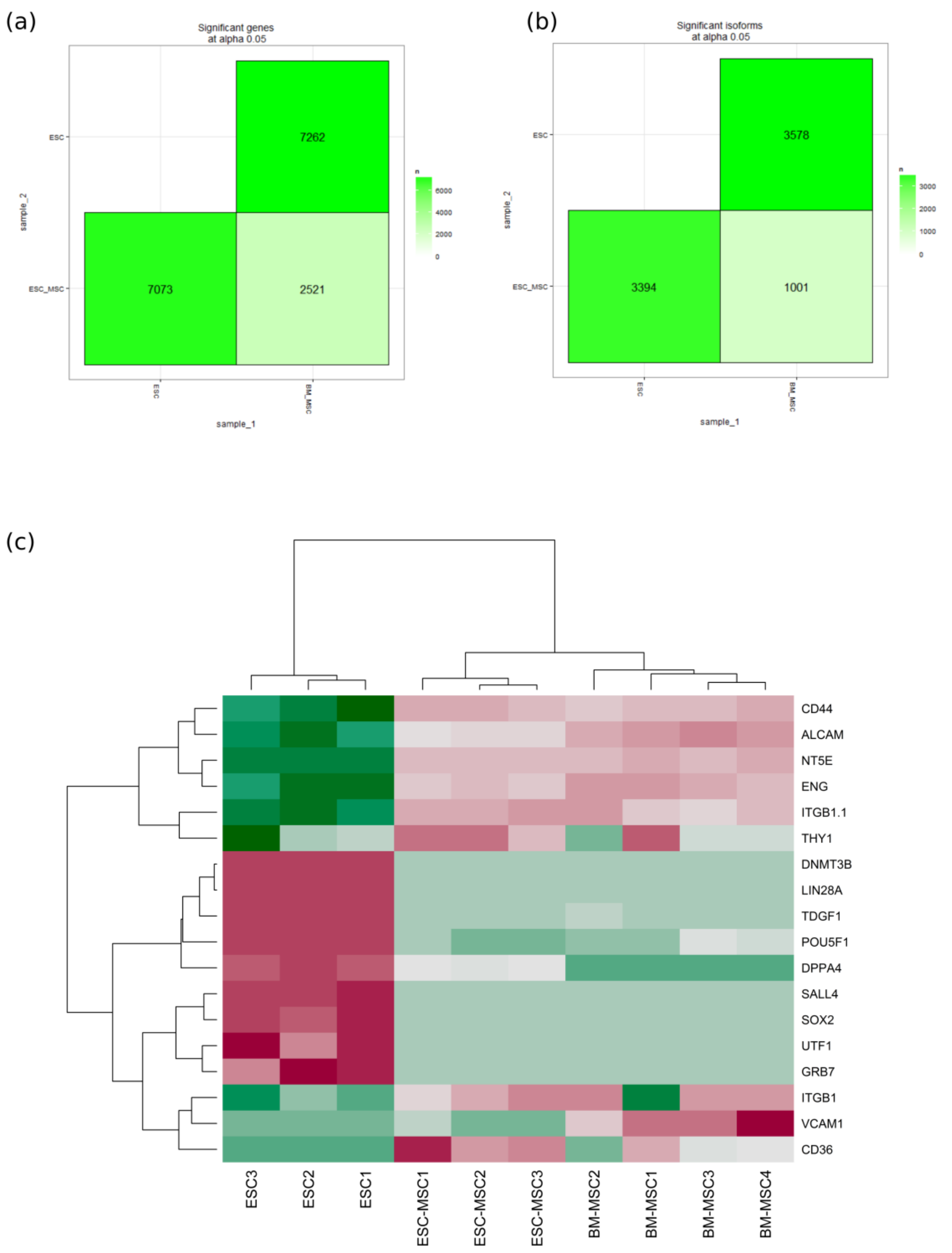

Supplementary Figure S2. Significant differentially expressed **(a)** genes and **(b)** isoforms (FDR < 0.05) based on RNA-seq for the following comparisons: ESC-MSC vs ESC, BM-MSC vs ESC and BM-MSC vs ESC-MSC. Plots were derived with the CummeRbund package. (c) Heatmap of ESC- and MSC-specific marker genes.

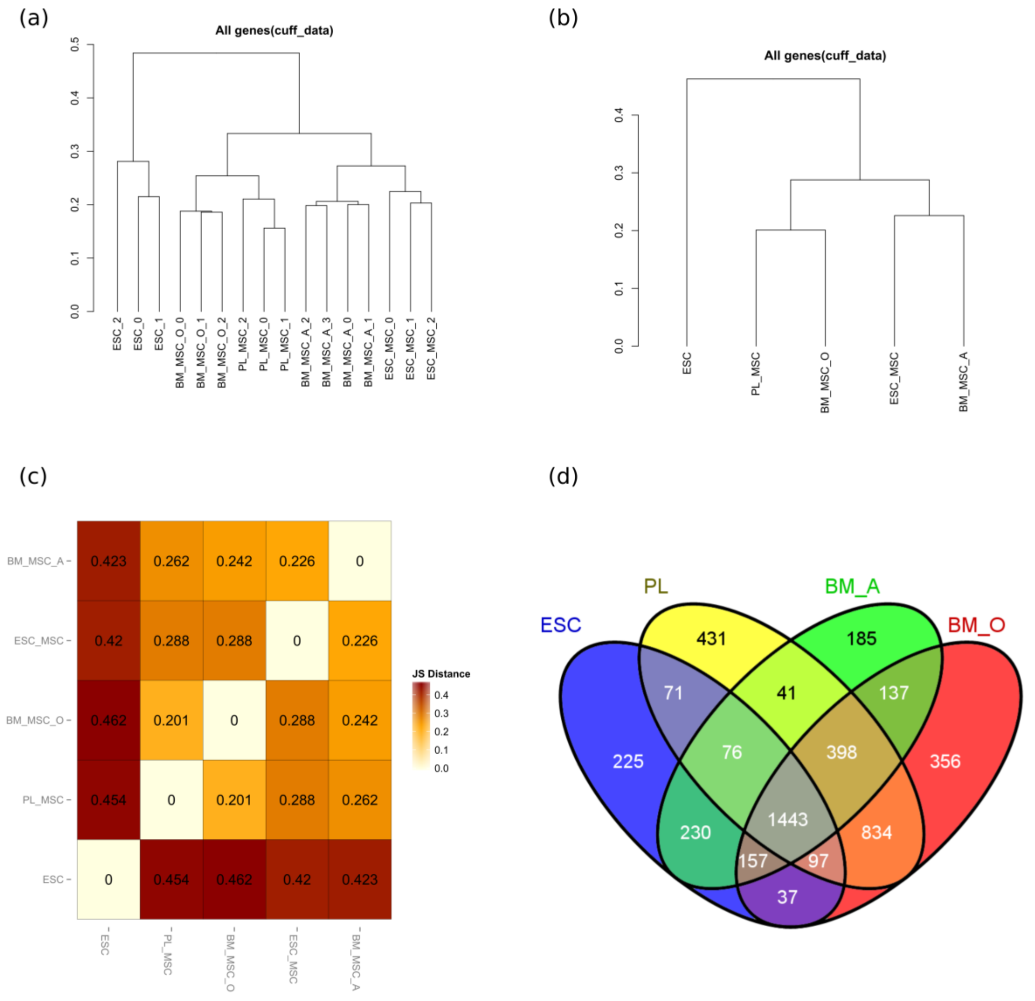

(e)

Comparison	GENE COUNT	
ESC / ESC-MSC	7446	
ESC / BM-MSC_A	7661	
ESC / PL_MSC	8500	
ESC / BM_MSC_O	8990	
ESC-MSC / BM-MSC_A	1925	▶ MSC populations Billing et al.
ESC-MSC / PL_MSC	4416	
ESC-MSC / BM-MSC_O	4696	
BM-MSC_A / PL_MSC	4170	
BM-MSC_A / BM_MSC_O	4134	
PL-MSC / BM-MSC_O	2056	▶ MSC populations Roson-Burgo et al.

Supplementary Figure S3. Comparison of RNA-seq data with other MSC study[1]. Both data sets were analyzed by the same bioinformatics pipeline (see materials and methods section). Dendrograms on **(a)** replicates and **(b)** groups show clear separation of ESC from MSC (placenta-derived MSC (PL_MSC), bone marrow-derived MSC (BM_MSC), ESC-derived MSC (ESC_MSC)). In both studies, fetal and adult MSC were compared. In our study: ESC-MSC vs BM-MSC (_A), by Roson-Burgo et al.: PL-MSC vs BM-MSC (_O). **(c)** Distance matrix generated by CummeRbund. **(d)** Venn diagram of up-regulated proteins (FDR < 0.05) in different MSC populations when compared to ESC. **(e)** Gene counts of all pairwise comparisons (FDR < 0.05).

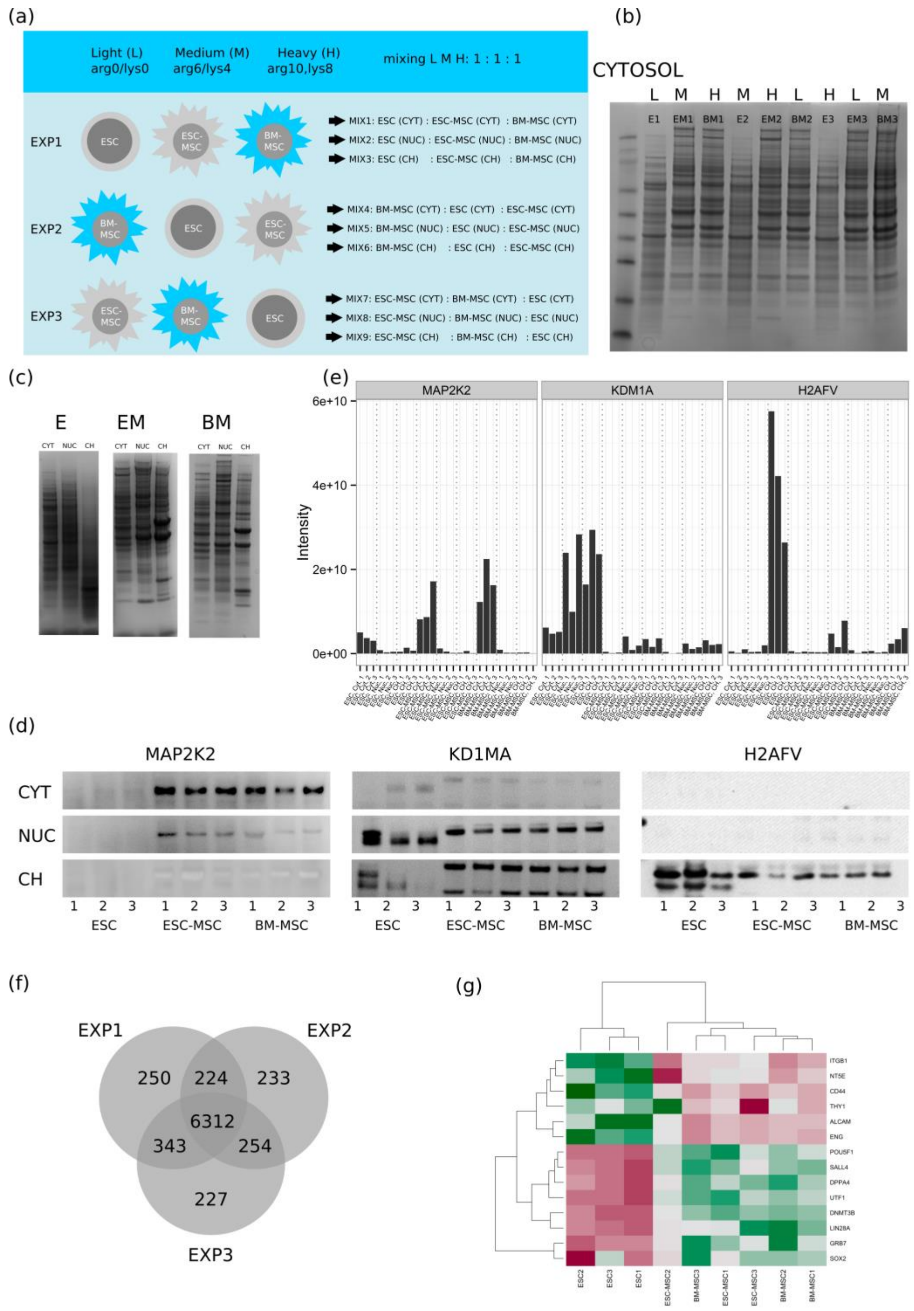

Supplementary Figure S4. Quantitative proteomics combined with SILAC labeling on ESC (E), ESC-MSC (EM) and BM-MSC (BM). (a) SILAC labeling schedule. SILAC labeling accounting for label swapping between ESC, ESC-MSC and BM-MSC. **(b)** As an example, SILAC-labeled cytosolic (CYT) protein extracts for E, EM and BM are shown for all three experiments. Protein extracts (10µg per lane) were separated by 4-12% SDS-PAGE. **(c)** Representative SDS-PAGE show cytosolic (CYT), nuclear (NUC) and chromatin-bound (CH) proteins for E, EM and BM. 10µg protein per lane was loaded. **(d)** Organelle markers confirm enrichment efficiency. Western blot analysis for three organelle markers (MEK1/2 for CYT, LSD1 for NUC/CH and H2AZ for CH) confirms protein extraction efficiency. 10 µg protein was loaded per lane. **(e)** Same markers were also measured by mass spectrometry presented as non-normalized intensity values. **(f)** Venn diagram of LC-MS/MS SILAC-quantified proteins per replicate. **(g)** Heatmap of pluripotency-associated factors (ESC-specific) and MSC-specific proteins measured by mass spectrometry.

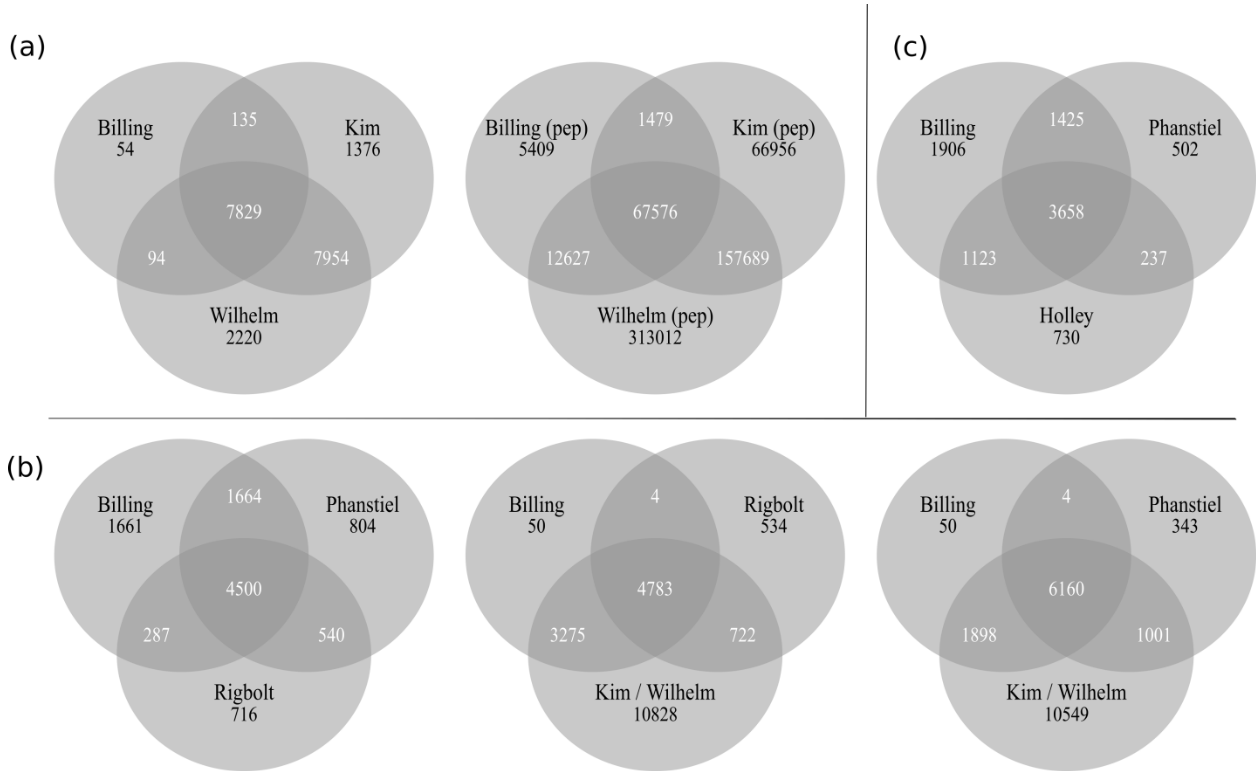

Supplementary Figure S5. Comparing presented proteomics data set with other studies. (a) Comparison with the human proteome maps[2,3] at protein and peptide level. **(b)** Comparison with the largest proteomics data sets available for ESC[4,5] at protein level. **(c)** Comparison with the largest proteomics data set available for MSC[6].

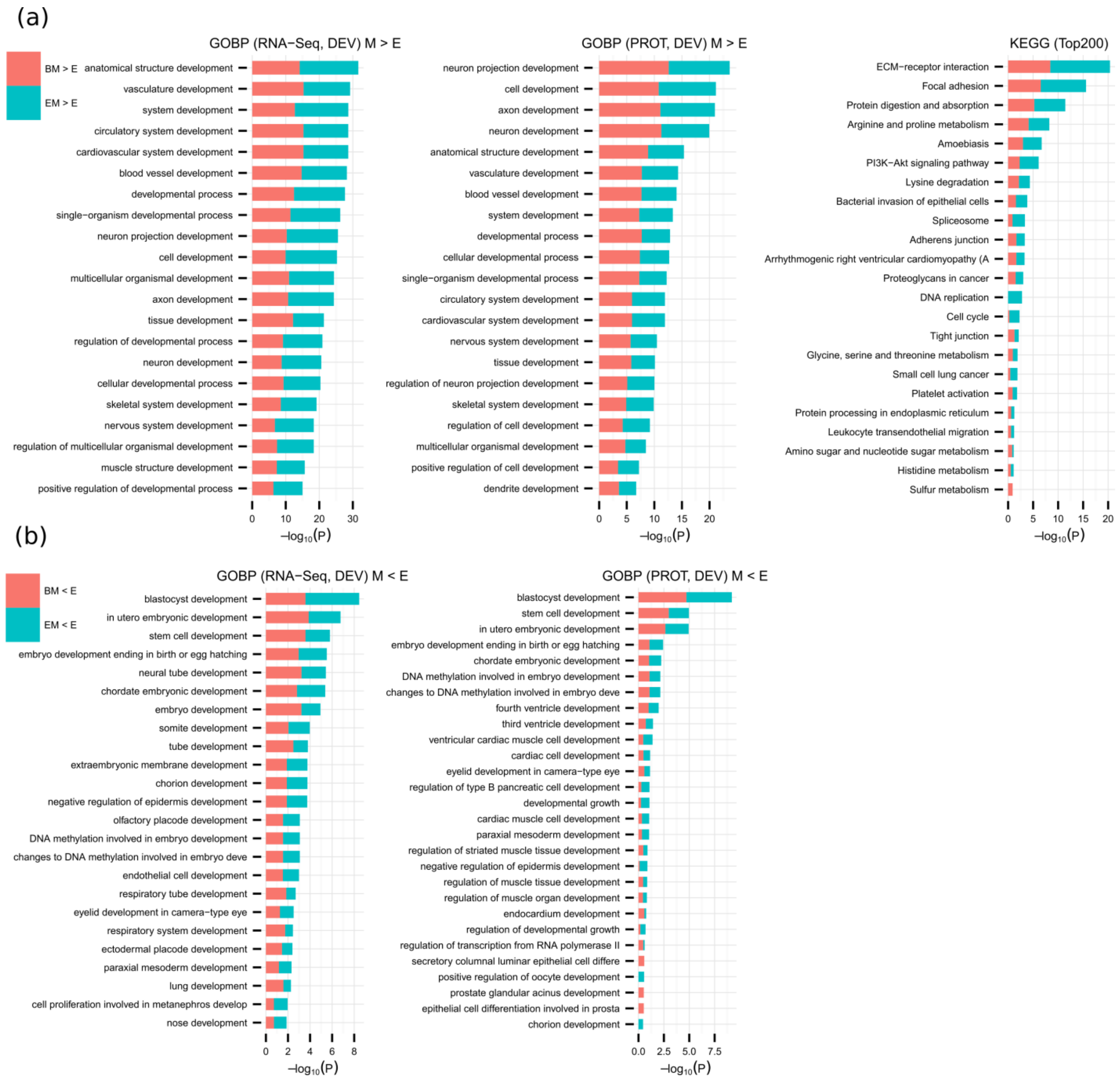

Supplementary Figure S6. Enrichment analysis for GOBP terms filtered for "development". Comparing MSC vs ESC with genes/proteins (a) up-regulated in MSC and **(b)** up-regulated in ESC. Enrichment was performed on RNA-seq (left panels) and proteomics data (right panels) using the comics package. Bar charts represent the most significant top 20 terms for each cell type sorted by the mean of $-\log_{10}$ p values.

Supplementary Table S3

The enrichment files can be downloaded using the following link:

https://bitbucket.org/billingetal2015c/disseminatebilling2015/downloads

REFERENCES

1. Roson-Burgo, B., Sanchez-Guijo, F., Cañizo, C. D. & Rivas, J. D. L. Transcriptomic portrait of human Mesenchymal Stromal/Stem cells isolated from bone marrow and placenta. *BMC Genomics* **15,** 910 (2014).
2. Kim, M.-S. *et al.* A draft map of the human proteome. *Nature* **509,** 575–581 (2014).
3. Wilhelm, M. *et al.* Mass-spectrometry-based draft of the human proteome. *Nature* **509,** 582–587 (2014).
4. Phanstiel, D. H. *et al.* Proteomic and phosphoproteomic comparison of human ES and iPS cells. *Nat. Methods* **8,** 821–827 (2011).
5. Rigbolt, K. T. G. *et al.* System-Wide Temporal Characterization of the Proteome and Phosphoproteome of Human Embryonic Stem Cell Differentiation. *Sci. Signal.* (2011). doi:10.1126/scisignal.2001570
6. Holley, R. J. *et al.* Comparative Quantification of the Surfaceome of Human Multipotent Mesenchymal Progenitor Cells. *Stem Cell Rep.* **4,** 473–488 (2015).
